# Mycotoxin Removal by *Lactobacillus* spp. and Their Application in Animal Liquid Feed

**DOI:** 10.3390/toxins13030185

**Published:** 2021-03-02

**Authors:** Chaima Ragoubi, Laura Quintieri, Donato Greco, Amel Mehrez, Imed Maatouk, Vito D’Ascanio, Ahmed Landoulsi, Giuseppina Avantaggiato

**Affiliations:** 1 Risques liés aux Stress Environnement aux, Lute et Prévention, Faculté des Sciences de Bizerte, Université de Carthage, Zarzouna 7021, Tunisia; chaymaragoubi9@gmail.com (C.R.); mehrezamel@yahoo.fr (A.M.); maatoukimed@yahoo.fr (I.M.); ahmed_landoulsi@yahoo.fr (A.L.); 2 Institute of Sciences of Food Production, National Research Council of Italy, 70126 Bari, Italy; laura.quintieri@ispa.cnr.it (L.Q.); donato.greco@ispa.cnr.it (D.G.); vito.dascanio@ispa.cnr.it (V.D.)

**Keywords:** mycotoxins, lactic acid bacteria, liquid feed, decontamination, adsorption, biodegradation, desorption

## Abstract

The removal of mycotoxins from contaminated feed using lactic acid bacteria (LAB) has been proposed as an inexpensive, safe, and promising mycotoxin decontamination strategy. In this study, viable and heat-inactivated *L. acidophilus* CIP 76.13T and *L. delbrueckii* subsp. *bulgaricus* CIP 101027T cells were investigated for their ability to remove aflatoxin B_1_ (AFB_1_), ochratoxin A (OTA), zearalenone (ZEA), and deoxynivalenol (DON) from MRS medium and PBS buffer over a 24 h period at 37 °C. LAB decontamination activity was also assessed in a ZEA-contaminated liquid feed (LF). Residual mycotoxin concentrations were determined by UHPLC-FLD/DAD analysis. In PBS, viable *L. acidophilus* CIP 76.13T and *L. delbrueckii* subsp. *bulgaricus* CIP 101027T cells removed up to 57% and 30% of ZEA and DON, respectively, while AFB_1_ and OTA reductions were lower than 15%. In MRS, 28% and 33% of ZEA and AFB_1_ were removed, respectively; OTA and DON reductions were small (≤15%). Regardless of the medium, heat-inactivated cells produced significantly lower mycotoxin reductions than those obtained with viable cells. An adsorption mechanism was suggested to explain the reductions in AFB_1_ and OTA, while biodegradation could be responsible for the removal of ZEA and DON. Both viable LAB strains reduced ZEA by 23% in contaminated LF after 48 h of incubation. These findings suggest that LAB strains of *L. acidophilus* CIP 76.13T and *L. delbrueckii* subsp. *bulgaricus* CIP 101027T may be applied in the feed industry to reduce mycotoxin contamination.

## 1. Introduction

Mycotoxins are harmful secondary metabolites produced by fungi that contaminate a wide range of food and feed products [1,2,3]. Depending on the frequency of occurrence and/or the severity of the disease that they produce, mycotoxins belonging to the aflatoxin, ochratoxin, and *Fusarium* toxin groups exert great impacts on animal/human health as well as on global trade [2,4]. Aflatoxin B_1_ (AFB_1_), zearalenone (ZEA), ochratoxin A (OTA), and deoxynivalenol (DON) are the most commonly occurring mycotoxins. They can cause a variety of diseases (mycotoxicoses) in a wide range of susceptible animal species [2,4]. Pigs are one of the most sensitive species to mycotoxins. These mycotoxins can cause both clinical and subclinical effects, including depressed food intake, impaired gut integrity, vaccination failure, liver overload, immune system interference, and damage to immune system cells, as well as acting as predisposing factors to infectious agents present on the farm. The presence of mycotoxins in feed can result in the poor performance of animals. The effects of mycotoxins depend on the health status, duration of exposure, nutrition, and co-occurrence of different toxins in the animals that consume them. When natural contamination with mycotoxins occurs, co-occurrence of more than one mycotoxin is likely to occur. According to a recent extensive global survey [5], almost 90% of feed and raw feed materials collected worldwide were found to be positive for at least one mycotoxin. Contamination with multiple mycotoxins was found in a large fraction of these samples (64%). Mycotoxin co-contamination depends mainly on the geographic region, climate, and weather conditions and can lead to the enhancement of the overall toxic effect of a single mycotoxin (synergistic effects) [6]. Therefore, despite most of the sample complying with the maximum levels/guidance values for mycotoxins in feed established in the European Union [7,8], the effect of mycotoxin co-occurrence on animal performance must be addressed [9,10]. Considering potential economic losses that mycotoxins can impose, highly efficient mycotoxin decontamination represents a crucial challenge. Several strategies, including physical, chemical, and biological methods, have been proposed to eliminate, inactivate, or reduce the mycotoxin concentration in food and feed commodities [11,12,13,14,15]. The application of traditional physical and chemical methods has some limitations concerning safety issues and losses in the nutritional value and palatability of feeds, coupled with limited efficacy and cost implications [12,16]. In contrast, biological decontamination involving the use of microorganisms or enzymatic extracts to facilitate biodegradation of the toxins and reduction of their absorption in the human and animal gastrointestinal tracts represents a sustainable approach that could be applied in the food and feed industries [11,13,14,17]. In addition, biological decontamination can be considered a suitable and valuable method for the decontamination of feed products intended to be used in the liquid state.

Liquid feed (LF) is a mixture of by-products from the liquid food industry and conventional dry materials or is made from dry raw materials mixed with water [18]. This alternative feeding strategy is widely used to improve animal health and performance, since it has several advantages, including simultaneous feed and water intake and positive effects on the animal gastrointestinal microflora [19,20,21,22,23]. LF manufacturing processes can also include fermentation with microorganisms, such as lactic acid bacteria (LAB), which have technological and functional properties that can improve feed safety and animal health by reducing the incidence of most common diseases [23,24,25]. However, as solid feed products, LFs can be contaminated by mycotoxins. High levels of mycotoxins have been detected in food by-products used as ingredients for food-based LFs [26,27,28]. The levels of DON and ZEA were found to exceed their limits in liquid pig feed [26].

LAB are typically associated with intestinal microflora and are also classified as probiotics due to their health effects in humans [29] and animals [30,31,32]. Many research reports have demonstrated their potential to either degrade mycotoxins or reduce their bioavailability in humans and animals [33,34,35]. The enteric bacteria *Lactobacillus rhamnosus* and *L. acidophilus*, as well as the plant-associated bacteria *L. plantarum*, *L. brevis,* and *L. sanfranciscensis* have been shown to have a high toxin decontamination ability [33,34,36]. To the best of our knowledge, few studies have reported on the reduction of mycotoxins in feed by LAB [37,38].

The aim of this work was to investigate the ability of some LAB strains isolated from healthy human urine (*L. acidophilus* CIP: 76.13T) and food products (*L. delbrueckii* subsp. *bulgaricus* CIP: 101027T) to reduce the main mycotoxins occurring in agricultural raw materials and food waste, i.e., AFB_1_, ZEA, OTA, and DON. In a preliminary study, one of these strains (*L. acidophilus* CIP: 76.13T) was found to be a promising probiotic due to its technological characteristics [39]. To evaluate the use of *L. acidophilus* CIP: 76.13T and *L. delbrueckii* subsp. *bulgaricus* CIP: 101027T in a decontamination strategy to minimize the risk of mycotoxin contamination of animal feed, both strains were assessed in a LF intended for pig feed.

## 2. Results and Discussion

### 2.1. Effect of Mycotoxins on LAB Growth

**Figure 1:**
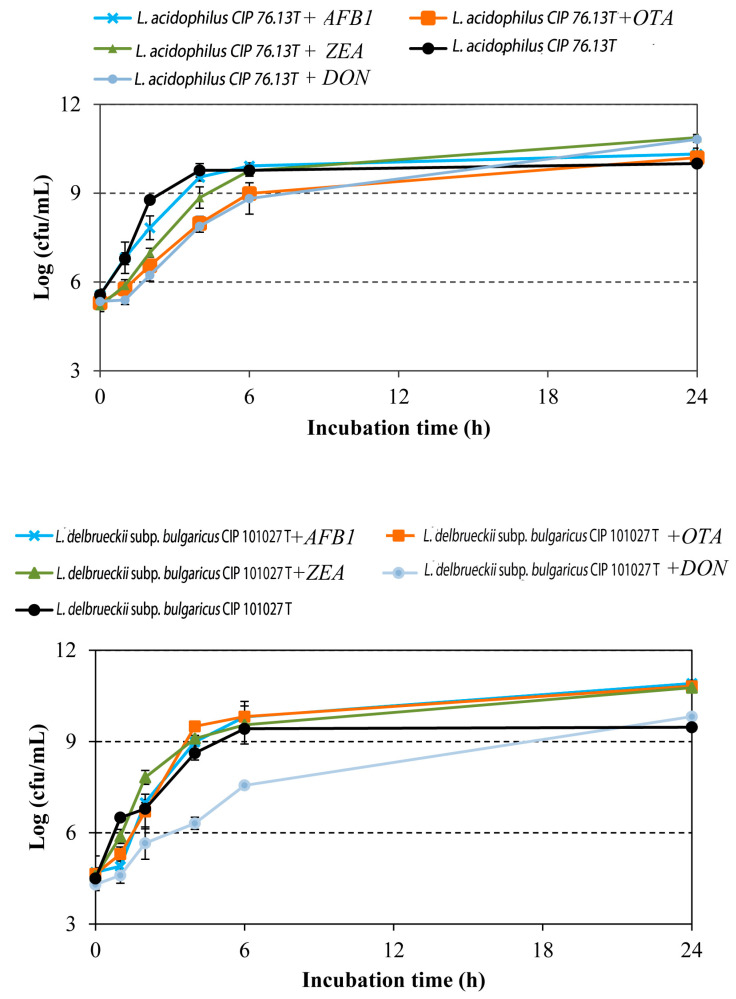
Growth curves of *L. acidophilus* CIP: 76.13T and *L. delbrueckii* subsp. *bulgaricus* CIP: 101027T obtained in MRS at 37 °C for 24 h in the absence or presence of target mycotoxins (aflatoxin B_1_ (AFB1), ochratoxin A (OTA), zearalenone (ZEA), and deoxynivalenol (DON)). The mycotoxin concentration was 1 μg/mL. Values are presented as means ± standard deviations (*n* = 3).

In order to assess the effect of mycotoxins on bacterial growth and viability, the growth of LAB was monitored in the presence and absence of the toxins, and the results were compared. As shown in Figure 1, the growth of *L. acidophilus* CIP: 76.13T was slightly inhibited by DON and OTA during the first 6 h of incubation (*p* < 0.05). In the presence of these mycotoxins, the microbial load of *L. acidophilus* CIP: 76.13T was reduced by ca. 1 log cfu/mL in comparison to that of the control samples (*p* < 0.003). Likewise, DON affected the growth of *L. delbrueckii* subsp. *bulgaricus* CIP: 101027T, causing a reduction in growth of 2 log cfu/mL after 6 h of incubation (*p* < 0.001) (Figure 1). After 24 h of incubation, no growth difference was registered when LAB strains were cultured in media supplemented (or not) with the mycotoxins. These findings agree with previous works showing that most mycotoxins do not inhibit the growth of *Lactobacillus* strains [40,41]. Recently, a dose-dependent inhibitory effect on the microbial load of *Lactobacillus* spp. was registered in mice gut microbiota after exposure to DON [42].

### 2.2. Mycotoxin Removal by LABs

In the current study, the ability of *L. acidophilus* CIP: 76.13T and *L. delbrueckii* subsp. *bulgaricus* CIP: 101027T to remove mycotoxins, i.e., AFB_1_, OTA, ZEA, and DON was assessed in two different media, MRS and PBS. In particular, PBS was used to evaluate the ability of LAB to reduce the concentrations of the targeted mycotoxins in the absence of a carbon source or other nutrients. 

The results of the microbiological analysis showed that the MRS microbial load reached an average level of 10 log cfu/mL after 24 h of incubation, while in PBS, a constant value was registered throughout the incubation period—5.5 log cfu/mL and 6 log cfu/mL (on average) at the beginning and the end of the assay, respectively. The final pH values in MRS and PBS were 4.2 and 6.0, respectively. As expected, bacterial growth in MRS was accomplished by a change in the pH value of the liquid medium caused by the formation of organic acids. No viable cells were found after the heat treatment of cell pellets.

As previously documented [43], mycotoxin removal by bacterial strains can occur in two ways: by biodegradation or transformation of the molecules and/or by adsorption into the cell walls of bacteria. To assess whether one or both mechanisms are involved in the removal of mycotoxins by our LAB strains, experiments using viable and thermally inactivated bacterial cells and desorption trials were carried out. Experimental results of mycotoxin removal expressed as percentages that were obtained using viable or inactivated *L. acidophilus* CIP: 76.13T and *L. delbrueckii* subsp. *bulgaricus* CIP: 101027T cells harvested in MRS and PBS are summarized in Table 1. (The related residual concentration values are also reported in Supplementary Table S1).

Except for ZEA, small reductions were registered for most mycotoxins (Table 1). The maximal OTA removal ability with viable cells was observed for *L. delbrueckii* subsp. *bulgaricus* CIP: 101027T (15%) in MRS, while OTA reduction values not higher than 8% were obtained when these cells were harvested in PBS. A slight increase in the removal activity of OTA was found after incubating heat-inactivated cells in PBS (*p* = 0.033). In this buffer, heat-inactivated *L. acidophilus* CIP: 76.13T and *L. delbrueckii* subsp. *bulgaricus* CIP: 101027T cells reduced the toxin concentration by 13% and 23%, with *L. delbrueckii* subsp. *bulgaricus* CIP: 101027T being the most efficient species (*p* = 0.033). Our results are in accordance with the outcomes of the study performed by Piotrowska [39], which examined the removal of OTA by three LAB, *L. plantarum*, *L. brevis*, and *L. sanfranciscensis*, in MRS and PBS, using viable or thermally inactivated bacterial cells. A study by Piotrowska [39] reports that OTA reduction was higher in thermally inactivated bacterial cells, suggesting that the toxin has a binding mechanism. Higher OTA adsorption by heat-inactivated rather than live cells in PBS was explained by changes occurring in the bacterial cell wall induced by high temperature [39]. Taking into account the study by Piotrowska [44], we suggest that our *L. acidophilus* CIP: 76.13T and *L. delbrueckii* subsp. *bulgaricus* CIP: 101027T strains can bind OTA to the surface structures of the cell wall. In addition to OTA, both LAB strains reduced AFB_1_ in MRS and PBS (*p* < 0.05) (Table 1) in a similar manner. In particular, after 24 h of incubation, viable *L. acidophilus* CIP: 76.13T and *L. delbrueckii* subsp. *bulgaricus* CIP: 101027T cells removed 34% and 31% of AFB_1_ in MRS and 6% and 16% in PBS (*p* = 0.024). As observed for OTA, the removal of AFB_1_ obtained by testing viable cells in MRS was significantly higher compared to in PBS (*p* < 0.001) (Table 1). This result may be related to the higher microbial concentration recorded in MRS than in PBS. In addition, in both MRS and PBS, AFB_1_ reductions obtained using thermally inactivated cells were comparable with those obtained using viable cells. Therefore, we suggest that adsorption is the mechanism that drives AFB_1_ removal by our strains. Cell wall binding of AFB_1_ by LAB is the most frequently described mechanism in the literature [35,45,46,47].

While adsorption seems to occur when OTA or AFB_1_ are incubated in contaminated liquid media inoculated with the herein tested strains, other mechanisms may be involved in the reduction of ZEA and DON.

As shown in Table 1, small removal values were recorded for DON when viable or heat-treated cells of *L. acidophilus* CIP: 76.13T and *L. delbrueckii* subsp. *bulgaricus* CIP: 101027T were tested in MRS, with these values being in the range of 3–9%. The strains did not differ in their ability to remove the toxin (*p* > 0.05). Higher values of DON reduction were obtained when viable or heat-inactivated cells from the strains were cultured in PBS. In this buffer, the *L. acidophilus* CIP: 76.13T and *L. delbrueckii* subsp. *bulgaricus* CIP: 101027T strains removed 31% and 30% of DON in the viable form and 14% and 19% in the heat-inactivated form (*p* < 0.001).

The percentage of ZEA removal by LAB strains ranged from 11 to 57%, depending on the culture medium and bacterial viability state. Similar to the results observed for DON, no significant difference was recorded between strains for their ZEA removal efficacy (*p* > 0.05). The highest ZEA removal values were registered in PBS by testing viable cells: 57% and 56% for *L. acidophilus* CIP: 76.13T and *L. delbrueckii* subsp. *bulgaricus* CIP: 101027T, respectively. In contrast, in MRS, for these strains and in the viable state, the average ZEA reduction value did not exceed 28%. Regardless of the medium and strain used, the efficacy of viable cells in reducing ZEA was significantly higher compared with heat-inactivated cells (*p* < 0.001). The level of ZEA removal by heat-inactivated cells was comparable in MRS and PBS, with values being 11–12% in MRS and 12–13% in PBS.

Taking into account the overall results for DON and ZEA reduction and considering that the removal of these mycotoxins in liquid media was significantly higher when viable cells of the LAB were tested or when a mineral buffer (PBS) was used where the mycotoxins were the only carbon source in the growth medium, we can assume that ZEA and DON are removed by a biodegradation mechanism. Our data agree with those reported by Chen et al. [48], who described LAB estereolitic activity as being based on ZEA degradation with no release of toxic metabolites. Regarding DON, the reduction percentage values registered for our strains fell within the range of those reported by other authors [49]. In addition, Garcia et al. [50] found that in PBS, the reduction in the DON concentration by LAB was due to the activity of extracellular enzymes that have not yet been identified. In order to confirm this mechanism and exclude the presence of an adsorption process on the cell walls of the strains, a desorption assay was performed for LAB cells incubated in PBS. These trials were performed for ZEA only, as this was selected as the most reduced toxin in our experimental conditions. Desorption trials are intended to determine the amount of toxin adsorbed onto bacterial cells and released when the adsorbent pellets are resuspended with an organic solvent. Indeed, in the case of mycotoxin reduction caused by an adsorption process, the solvent used for the desorption assay extracts the toxin from the pellet, and then its presence in the supernatant can be determined following centrifugation [51]. In our study, a mixture containing acetonitrile and water (9:1 *v*/*v*) was used to release ZEA from the adsorbent pellets of LAB, being the most commonly used solvent for ZEA analysis in food and feed samples. Whether other mechanisms, such as metabolization, underlie the reduction of ZEA by LABs, it cannot be detected in the supernatants.

The results of the desorption study are presented in Table 2. Experimental values expressed as percentages refer to the amount of toxin released by the solvent with respect to the amount of ZEA reduced in PBS. As described above (Table 1), cells from viable *L. acidophilus* CIP: 76.13T and *L. delbrueckii* subsp. *bulgaricus* CIP: 101027T strains reduced ZEA by 57% and 56% in PBS (*p* < 0.001). A negligible amount of toxin was released when the adsorbent pellets from these bacteria were treated with the organic solvent. For both LAB strains, the amount of ZEA desorbed from viable cells did not exceed 2% (*p* < 0.001) (Table 2). In contrast, heat-treated cells from *L. acidophilus* CIP: 76.13T and *L. delbrueckii* subsp. *bulgaricus* CIP: 101027T strains reduced the toxin concentration by only 13% and 12% in PBS (*p* < 0.001), respectively, and almost all of the adsorbed toxin was released. For these strains, the desorption values were 56% and 83%, respectively. Based on the results obtained from adsorption and desorption trials, we can conclude that both mechanisms, biodegradation and adsorption of ZEA, can occur when the LAB strains are incubated with the toxin in PBS, depending on their state. In particular, viable cells from the strains reduce the toxin by biodegradation, especially in a medium in which ZEA is the only source of carbon. Therefore, the toxin cannot be recovered from the cell pellets in desorption trials. In addition, a lower amount of toxin can be reduced by adsorption, which occurs when cells are killed by heat treatment.

Several studies have reported the ability of LAB to reduce the mycotoxin concentration by adsorption and enzymatic degradation processes [33], which are both promising approaches for the decontamination of mycotoxin-contaminated foodstuffs. However, bacterial biodegradation of mycotoxins is believed to be the best solution to prevent the toxic effects of mycotoxins, since the possibility of release of the bound mycotoxins from the cell wall is an inherent disadvantage of the microbial adsorption mechanism which cannot be disregarded. As underlined by Chen et al. [48], microbial biodegradation of ZEN as a means of food and/or feed detoxification has not been studied extensively. This may be due to the lack of information concerning the mechanisms of biodegradation, the toxicity of the degradation products, and the safety of the microorganisms for animals. Recently, esterase activity by selected LAB strains belonging to the *L. plantarum* species was demonstrated to play a role in ZEA removal [48]. Metabolites produced by these hydrolytic enzymes act as substrates for metabolic pathways, sustaining microbial survival in PBS medium. Regarding ZEA removal, no related metabolites (i.e., α- and β-zearalenol, zearalanone and its reduced metabolites α- and β-zearalanol) were detected in inoculated MRS and PBS at the end of the incubation period. Although the mechanism of mycotoxin removal and the optimization of culture conditions for microbial growth need deeper investigation, the application of our strains, preferably as viable cells, in the preparation of animal feed may help to counteract the toxic effects of mycotoxins and to promote additional probiotic properties responsible for maintaining a healthy gastro-intestinal tract. Indeed, one of our LAB strains, *L. acidophilus* CIP 76.13, was previously investigated for its technological and probiotic properties; in particular, it exhibited high proteolytic and lipolytic activities and tolerance at several pH values (pH 3, 5, 7) [39].

### 2.3. ZEA Removal in Animal Liquid Feed

Taking into account the overall results of the study, the *L. acidophilus* CIP: 76.13T and *L. delbrueckii* subsp. *bulgaricus* CIP: 101027T strains were further tested to assess their efficacy to reduce ZEA in a liquid feed model.

The recovery and repeatability of the method used to analyze ZEA in LF were determined by spiking blank LF with different concentrations of the toxin. As shown in Table 3, this method was found to be satisfactory as it provided a recovery level of 92% for the highest mycotoxin concentration (1 µg/mL) and 81% for the lowest one (0.1 µg/mL). The within-day precision expressed as the relative standard deviation (RSD_r_) was also satisfying, being lower than 7%.

*L. acidophilus* CIP: 76.13T and *L. delbrueckii* subsp. *bulgaricus* CIP: 101027T strains grew well in LF, reaching concentrations of 7.5 and 9.5 log cfu/mL after 24 h of incubation at 37 °C (Table 4). The percentages of ZEA removed by *L. acidophilus* CIP: 76.13T after 24 and 48 h of incubation were 14% and 22% (*p* = 0.016), respectively. *L. delbrueckii* subsp. *bulgaricus* CIP: 101027T showed the same trend for toxin removal (10% reduction after 24 h and 23% after 48 h) (*p* = 0.004) (Table 4 and Supplementary Table S2).

The level of efficacy of our LAB strains in removing ZEA in a complex environment, such as from a feed in liquid form, was comparable to that recorded in MRS. However, ZEA removal in MRS or LF was lower than the level of toxin reduction obtained with PBS. This may be explained by the interfering processes exerted by some components of the culture media. In addition, the negative effect of the final pH of bacterial cultures could have also played a role. As observed when MRS or LF was used as the growth media, at the end of the incubation period, bacterial cultures reached pH values lower than 5. Many studies have shown that culture conditions, such as the temperature, pH, incubation time, bacterial biomass, and growth medium, significantly affect mycotoxin removal by LAB [33]. Hsu et al. [52] demonstrated that the ability of the *B. licheniformis* strain CK1 to remove ZEA increased when the pH of the medium increased from 2.5 to 6.0. Wang et al. [53] also confirmed that ZEA removal by *Lysinibacillus* spp. is strictly dependent on several growth parameters, including pH. In particular, a reduction in ZEA of close to 100% was observed at 37 °C and pH 7.0. Further investigations are required to determine the optimal experimental conditions for ZEA removal by our viable LAB strains. 

To the best of our knowledge, this is the first study to investigate the mycotoxin decontaminating activity of LAB strains in a LF model. Mycotoxins in LF can originate from contaminated raw feed materials used to prepare liquid formulations [28,54,55]. Few studies in the literature have addressed the use of LAB to reduce the mycotoxin concentration in food or feed. Some LAB have been found to effectively reduce the ZEA concentration by more than 50% in maize meal and maize porridge after 4 days of incubation [56,57]. A significant amount of α-zearalenol was found after 24 h of incubation with different LAB species in a simulated corn silage model contaminated with ZEA [58], indicating that ZEA removal may lead to the formation of more toxic metabolites. ZEA metabolites (i.e., α- and β-zearalenol, zearalanone, and α- and β-zearalanol) were not detected in contaminated LF samples inoculated with our LAB strains.

## 3. Conclusions

This study examined the ability of two potential probiotic LAB strains, *L. acidophilus* CIP: 76.13T and *L. delbrueckii subsp. bulgaricus* CIP: 101027T, to remove mycotoxins (OTA, ZEA, DON, AFB_1_) in in vitro experiments and in an animal LF used as a feed model. An experimental procedure to study the mechanism of mycotoxin removal (adsorption or metabolism) by bacterial strains is presented. In particular, mechanisms involved in toxin decontamination by LAB strains were investigated by monitoring toxin reduction under different growth conditions (different media and presence or absence of nutrients) and in a different physiological state (viable or heat-inactivated cells), and by performing desorption trials (for ZEA only). As expected, the reduction percentage differed depending on the experimental conditions. In general, the mycotoxin removal ability observed for viable cells was higher compared with that obtained with heat-inactivated cells. For both strains, the highest reduction values were registered by viable cells for AFB_1_ in MRS (by ca. 30%) and for ZEA and DON in PBS (by ca. 57 and 30%, respectively). In contrast, the OTA concentration was not significantly affected. A binding mechanism is suggested to be responsible for OTA and AFB_1_ removal, while biodegradation may be responsible for reductions in DON and ZEA. A moderate level of ZEA removal was also found after 24 and 48 h of incubation in animal LF. 

Although the mechanism of mycotoxin removal and the optimization of culture conditions for microbial growth need deeper investigation, the application of viable LAB cells with probiotic potential, in particular, *L. acidophilus* CIP: 76.13T, to the preparation of animal feed may help to counteract the toxic effects of mycotoxins and contribute to the maintenance of a healthy gastrointestinal tract.

## 4. Materials and Methods

### 4.1. Chemicals and Reagents

All chemicals used were of analytical grade. All solvents (HPLC grade) were purchased from J.T. Baker (Deventer, The Netherlands). Water was of MilliQ quality (Millipore, Bedford, MA, USA). Mycotoxin standards of AFB_1_, ZEA, OTA, and DON (purity >99%) were purchased from Sigma Aldrich (Milan, Italy). Culture media were purchased from Oxoid S.p.A. (Garbagnate, Milan, Italy) unless otherwise specified.

Mycotoxin stock solutions of AFB_1_, OTA, ZEA, and DON (1 mg/mL) were prepared by dissolving solid commercial toxins in acetonitrile (HPLC grade) and stored in the dark at 4 °C. The mycotoxin solutions used for the decontamination experiments and the calibrants used for the UHPLC analysis were prepared by diluting each stock solution in MRS (Merck, Germany), PBS, or UPLC mobile phase before use.

### 4.2. Bacterial Strains and Culture Conditions

*L. acidophilus* CIP: 76.13T and *L. delbrueckii* subsp. *bulgaricus* CIP: 101027T isolated from human urine and yogurt, respectively, were obtained from the Collection of Institute Pasteur (CIP; Paris, France). Strains were grown overnight on MRS at 37 °C under microaerophilic conditions and shaken with 125 strokes/min unless otherwise mentioned. Fresh cultures were transferred in Nutrient Broth (NB, BioLifeItaliana, Milan, Italy) containing 20% glycerol (*w*/*v*) and stored at −80 °C until subsequent experiments.

### 4.3. Effect of Mycotoxins on Microbial Growth 

Each strain was grown overnight in 5 mL of MRS at 37 °C under aerobic conditions and agitation (150 strokes/min). Fresh cultures were inoculated in MRS and incubated at 37 °C to reach the optical density value at 600 nm (OD600) of 1.0 ± 0.07 (corresponding to 9.1 ± 0.8 log cfu/mL, on average; mid-exponential growth phase). After dilution in sterile saline solution (0.95% NaCl), each culture was inoculated in triplicate at 5.03 ± 0.75 log cfu/mL (on average) in MRS containing 1 µg/mL of each mycotoxin and incubated at 37 °C under agitation (150 strokes/min) for 1, 2, 3, 4, 6, and 24 h. At each incubation time, samples were analyzed for microbial enumeration by plating serial decimal dilutions onto MRS agar. Plates were incubated at 37 °C for 24 h under conditions of aerobiosis. 

### 4.4. Mycotoxin Removal by Viable and Heat Inactivated LAB Cells 

Overnight LAB cultures, cultivated as described above, were inoculated (1%) in 5 mL of MRS and incubated at 37 °C to reach the OD600 value of 0.6, corresponding to 5–6 log cfu/mL. After that, the cultures were harvested by centrifugation (13,000 rpm for 5 min at 4 °C) and washed twice in sterile saline solution (0.9% *w*/*v* NaCl). 

To perform mycotoxin removal experiments using viable cells, the pellets were resuspended in 5 mL of MRS or sterile PBS supplemented with 1 µg/mL of each mycotoxin and incubated for 24 h, as previously described. At the start and the end of the incubation period, the pH of cultures was determined with the Beckman Coulter Φ 340 pH/TempMeter system (Fullerton, CA, USA). After the incubation period, samples were centrifuged for 10 min at 13,000 rpm. 

In addition, mycotoxin reduction experiments were performed using inactivated LAB strains. LAB cells were inactivated as described by Piotrowska [44]. Briefly, LAB suspensions in PBS (6 log cfu/mL) were autoclaved for 15 min at 121 °C under 1 atm of pressure. Then, cells were recovered after centrifugation and resuspended in PBS or MRS and tested with mycotoxin removal experiments, as described above. 

Mycotoxin removal experiments were performed in triplicate, and microbial loads were determined by plating decimal dilutions on MRS agar at the beginning and end of the incubation period. For each experiment, a control treatment without a bacterial strain (blank control) was subjected to the same test procedure and used as the background control during the analysis to investigate the stability of mycotoxins in MRS or PBS.

To measure the level of mycotoxin removal by each strain, supernatants of liquid cultures were analyzed by UPLC-FLD/PDA methods after a clean-up step using immunoaffinity columns from VICAM^©^ (Watertown, MA, USA). Briefly, 0.5 mL of each supernatant was diluted with PBS (1.5 mL) and cleaned up using an immunoaffinity column at a flow rate of about 1 drop per second. The column was first washed with 5 mL of PBS containing Tween 20 (0.01%, *v*/*v*), followed by 5 mL of water. ZEA and DON were eluted with 2 mL of methanol in a 4 mL silanized amber vial. The eluates were dried at 50 °C under an air stream, and the residues were re-dissolved with 250 μL of a mixture containing methanol and water (20:80, *v*/*v*) prior to UPLC injection. AFB_1_ and OTA were collected from the immunoaffinity columns with 1.5 mL of methanol. After the addition of 1.5 mL of water, samples were injected into the LC system. Mycotoxins were analyzed by UPLC using the method presented by Greco et al. [59] and Adunphatcharaphon et al. [60]. In addition, a UPLC-PDA method was used for the simultaneous determination of ZEA and its derived metabolites (α- and β-zearalenol, α- and β-zearalanol, zearalanol). The apparatus was an ACQUITY UPLC™ system (Waters, MA, USA) combined with a PDA detector (PDA ACQUITY UPLC^®^). The toxins were separated on a UPLC column (UPLC^®^ BEH C18 column, 2.1 × 100 mm, 1.7 µm) preceded by an in-line filter (0.2 µm) under a gradient program. Water (solvent A) and methanol (solvent B) were used as mobile phases, with a flow rate of 0.35 mL/min. The gradient elution started with 15% B, which was linearly increased to 100% in 23 min. After elution, the column was re-equilibrated with 15% methanol for 10 min. The column and the samples were maintained at constant temperatures of 50 and 15 °C, respectively. The injection volume was 10 µL. The UV absorption spectra of toxins were recorded in the range of 190–400 nm. UV absorbance data were collected with a bandwidth of 1.2 nm and without digital filtering at a wavelength of 220 nm.

The concentration of mycotoxins removed from liquid media was calculated as the difference between the concentration of mycotoxins in the supernatant of blank tubes with no LAB and the concentration found in the supernatant of the experimental tubes with LAB. This amount was then related to the quantity present in the supernatant of blank tubes and expressed as a percentage.

### 4.5. Mycotoxin Desorption Experiments

Desorption experiments were performed to determine the amount of toxin adsorbed onto the bacterial cells and released using an organic solvent. These tests allowed us to assess whether mycotoxin reduction by viable or inactivated LAB cells was due to an adsorption process or other mechanisms. The desorption study was performed for ZEA only, as this was the mycotoxin removed to the greatest extent by the tested LAB strains. First, the amount of ZEA removed by the bacterial pellets (containing viable or heat-inactivated cells) was measured. LAB suspensions of each strain (5–6 log cfu/mL) in PBS were incubated for 24 h in the presence of 1 µg/mL of the toxin. After incubation, the suspensions were centrifuged for 20 min at 14,000 rpm. The supernatants were completely removed and analyzed to determine their mycotoxin contents, as described above, to measure mycotoxin removal. The pellets were resuspended with 1 mL of a mixture containing acetonitrile and water (90:10, *v*/*v*) and incubated at 37 °C for 60 min with soft agitation (150 stroke/min). At the end of the incubation period, samples were centrifuged, and 500 µL of supernatant was dried under an air stream at ca. 50 °C and reconstituted with 500 µL of a mixture containing water and methanol (85:15, *v*/*v*) before chromatographic injection. Desorption studies were performed in triplicate. To measure mycotoxin desorption, the amount of toxin released from the adsorbing cell pellets by the organic solvent was compared with the amount removed by the pellets from PBS and then expressed as a percentage.

### 4.6. Mycotoxin Removal by LAB from Animal LF

Taking into account the results of the preliminary study showing a 50% reduction of ZEA by LAB, the removal of this mycotoxin was investigated in a LF intended for weaning piglets. The main ingredients for the LF were kindly provided by the New Feed Team S.r.l. (Lodi, Italy): maize silage (pH 4.5; 35% moisture) and dried feed (maize, soy, cereal bran vitamin and mineral mixture). The LF was prepared, as suggested by the New Feed Team S.r.l., by mixing the maize silage (10%, *w*/*v*) with the dried feed (10%, *w*/*v*) in distilled water (80%, *w*/*v*) and stirring for 20 min at room temperature. Then, the mixture was autoclave-sterilized at 120 °C for 15 min. After sterilization, the mixture was supplemented with sterilized water (25%; *v*/*v*) and stirred vigorously, to give a soft mixture for mycotoxin removal microbial tests. The latter steps (autoclave-sterilizing, water supplementation and vigorous stirring) were required to remove naturally occurring microorganisms and to prepare a suitable LF for bacterial growth. ZEA reduction experiments were performed in sterile Falcon^®^ 12-welled polystyrene microplates (BD Biosciences, Erembodegem, Belgium). Sterilized LF containing 1 μg/mL of ZEA was inoculated with ca. 6 log cfu/mL of each LAB strain. A volume (2 mL) of both inoculated and un-inoculated LF was dispensed into the 12-welled plate and fermented for 24 and 48 h at 37 °C under aerobiosis and static conditions. Uninoculated LF supplemented with the toxin was used as a background control to investigate the stability of ZEA in LF and its unspecific binding to the components of the LF matrix. Test samples and controls were prepared as six independent replicates to be used for microbiological and chemical analyses (3 replicates each). 

To assess LAB growth in LF, the microbial load of the inoculated samples containing the toxin (or not) was measured after each incubation period. Thus, 2 mL of each LF sample was diluted with 18 mL of sterile saline solution (0.9% NaCl), and these samples were homogenized and decimally diluted in sterile saline solution before plating on MRS agar (24 h, 37 °C under aerobiosis) for enumeration. 

To measure the removal of ZEA from contaminated LF by LAB strains, the toxin was analyzed according to the official method (ZEA: ISO 17372:2008) with a few changes [61]. Briefly, the LF cultures (2 mL each) were extracted in 50 mL Erlenmeyer flasks using 10 mL of a mixture containing water and acetonitrile (10:90, *v*/*v*). The flasks were incubated for 1 h at room temperature under soft agitation (150 stroke/min). Then, the extracts were filtered using Whatman^®^ filter papers (grade 4), and 1 mL of each extract was diluted with 4 mL of water. To clean up the samples, 1 mL of each diluted extract was passed through a ZearalaTest^TM^ immunoaffinity column. After the washing step, the toxin was eluted with 2 mL of methanol. Cleaned-up samples in methanol were dried under an air stream at 50 °C, reconstituted with a mixture containing water and methanol (85:15, *v*/*v*), and analyzed by UHPLC.

In order to check the ability of the method to analyze ZEA in LF samples, recovery and repeatability were determined by spiking LF with ZEA at two levels (0.1 and 1 µg/mL) and analyzed as six independent replicates. These toxin levels were chosen to ensure the reliable analysis of mycotoxins in LF when a 90% reduction occurred. The concentrations of ZEA recovered from LF samples were determined by using a standard calibration curve. Repeatability was expressed as the relative standard deviation, RSD_r_ (*n* = 6).

### 4.7. Statistical Analysis

The effect of mycotoxins on bacterial growth was monitored in the presence and absence of each toxin, and the results were compared by using one-way ANOVA and the Dunnett test as post hoc analyses. The mycotoxin removal by LAB strains is expressed as the mean ± standard deviation of three independent replicate experiments. Data were statistically analyzed by two-way ANOVA using statistical analysis software (Statistica 12.0, StatSoft^®^). Two-way ANOVA was used to examine the influences of the medium and strains present on mycotoxin reduction. Post-hoc analyses of sample subgroups were performed using the Duncan test as a multiple comparison procedure. The significance level was set at 0.05.

## Figures and Tables

**Table 1:** Percentages of mycotoxin reduction by viable (6 log cfu/mL; VC, viable cells) and heat-inactivated cells (6 log cfu/mL; HIC, heat-inactivated cells) from *L. acidophilus* CIP: 76.13T and *L. delbrueckii* subsp. *bulgaricus* CIP: 101027T strains obtained in MRS and PBS after 24 h of incubation at 37 °C under aerobic conditions. The initial concentration of zearalenone (ZEA), deoxynivalenol (DON), aflatoxin B1 (AFB_1_), and ochratoxin A (OTA) in MRS and PBS was 1 µg/mL in all cases.

Mycotoxin Reduction (%)								
LAB Strain	ZEA		DON		AFB_1_		OTA	
	MRS	PBS	MRS	PBS	MRS	PBS	MRS	PBS
VC *L.* *acidophilus* CIP: 76.13T	28.3 ± 1.8 ^ay^	57.4 ± 3.4 ^az^	8.8 ± 1.2 ^ay^	30.5 ± 2.6 ^az^	33.5 ± 3.1 ^ay^	6.3 ± 2.7 ^az^	7.1 ± 3.8 ^ay^	8.3 ± 2.3 ^ay^
VC *L.* *delbrueckii* subsp. *bulgaricus* CIP: 101027T	28.5 ± 1.8 ^ay^	56.4 ± 4.2 ^az^	5.4 ± 0.8 ^aby^	30.0 ± 0.5 ^az^	30.8 ± 3.6 ^ay^	15.9 ± 1.4 ^bz^	15.3 ± 2.5 ^by^	4.0 ± 1.2 ^az^
HIC *L.* *acidophilus* CIP: 76.13T	11.9 ± 1.8 ^by^	12.8 ± 4.3 ^by^	3.1 ± 0.8 ^by^	14.1 ± 1.1 ^bz^	27.4 ± 1.9 ^aby^	11.9 ± 2.4 ^bz^	2.2 ± 2.5 ^cy^	13.2 ± 1.0 ^bz^
HIC *L. delbrueckii* subsp. *bulgaricus* CIP: 101027T	10.7 ± 1.8 ^by^	11.7 ± 3.8 ^by^	6.8 ± 2.4 ^aby^	19.4 ± 4.8 ^bz^	22.8 ± 0.5 ^by^	14.3 ± 2.4 ^bz^	1.1 ± 0.9 ^cy^	22.5 ± 3.4 ^cz^

Results are mean values ± standard deviations of three independent experiments. A statistical analysis was performed considering all data obtained for each mycotoxin. For each mycotoxin, comparisons were performed between MRS and PBS and between strains. ^a–c^ Different superscript letters in the same column (medium) indicate a significant effect between strains. ^y–z^ Different superscript letters in the same row (strain) indicate a significant effect between media (ANOVA, Duncan test with *p* < 0.05 representing significance).

**Table 2:** Percentages of zearalenone (ZEA) desorbed from viable cells (VC) and heat-inactivated cells (HIC) of the *L. acidophilus* CIP: 76.13T and *L. delbrueckii* subsp. *bulgaricus* CIP: 101027T strains. Desorption data are given as means ± standard deviations of three independent experiments.

LAB Strain	ZEA Desorbed (%)	
	VC	HIC
*L. acidophilus* CIP: 76.13T	1.9 ± 0.5 ^a^	55.5 ± 2.2 ^b^
*L. delbrueckii* subsp. *bulgaricus* CIP: 101027T	1.9 ± 0.2 ^a^	82.9 ± 5.4 ^c^

Values labeled with different superscript letters are significantly different (ANOVA, Duncan test with *p* < 0.05 representing significance).

**Table 3:** Results of the validation study of the analytical method used to assess the zearalenone (ZEA) concentration in an animal liquid feed (LF). Mean recoveries and within-day relative standard deviations were obtained from spiked samples that were analyzed six times at each spiking level.

Spiking Level (µg/mL)	Recovery % (RSD_r_, %)
0.1	81 (7)
1.0	92 (6)

**Table 4:** Zearalenone (ZEA) removal by *L. acidophilus* CIP: 76.13T and *L. delbrueckii* subsp. *bulgaricus* CIP: 101027T determined after 24 and 48 h of incubation in animal liquid feed (LF) at 37 °C. The microbial count at 0, 24, and 48 h is also reported. Values represent means ± standard deviations of three independent experiments.

LAB Strain	ZEA Reduction (%)		Log cfu/mL		
	24 h	48 h	0 h	24 h	48 h
*L. acidophilus* CIP: 76.13T	14 ± 2 ^a^	22 ± 2 ^b^	5.6 ± 0.1 ^a^	7.5 ± 0.1 ^b^	5.6 ± 0.8 ^a^
*L. delbrueckii* subsp. *bulgaricus* CIP: 101027T	10 ± 2 ^a^	23 ± 2 ^b^	5.8 ± 0.1 ^a^	9.5 ± 0.6 ^c^	8.9 ± 1.1 ^d^

Values within each group with different superscript letters are statistically different (ANOVA, Duncan test with *p* < 0.05 representing significance).

## Data Availability

Not applicable.

## Supplementary Materials

The following are available online at https://www.mdpi.com/2072-6651/13/3/185/s1, Table S1: Residual mycotoxin concentration (µg/mL) in supernatant samples obtained by incubating viable (VC) and heat inactivated cells (HIC) of *L. acidophilus* CIP: 76.13T and *L. delbrueckii* supp. *bulgaricu* s CIP: 101027T with 1 µg/mL of each mycotoxin. Mycotoxin removal by LAB strains was determined in MRS and PBS after 24 h of incubation at 37 °C under aerobic conditions. AFB1: Aflatoxin B1, OTA: Ochratoxin A, ZEA: Zearalenone, DON: Deoxynivalenol., Table S2: Residual ZEA concentration in liquid feed (LF) obtained incubating viable cells of *L. acidophilus* CIP: 76.13T and *L. delbrueckii* supp. *bulgaricus* CIP: 101027T with 1 µg/mL of the toxin. ZEA removal in LF by LAB strains was determined after 24 and 48 h of incubation in LF at 37 °C. Microbial count at 0, 24 and 48 h is also reported. Values represent means ± standard deviations of three independent experiments.
