# Supplementary material for: Mycotoxin Removal by Lactobacillus spp. and Their Application in Animal Liquid Feed
Source: Toxins (Basel). 2021 Mar 2;13(3):185. doi: 10.3390/toxins13030185 (PMC8000088; doi:10.3390/toxins13030185)
Supplement: Supplementary file 1 [file toxins-13-00185-s001.pdf]

Supplementary Materials: Mycotoxin Removal by *Lactobacillus* spp. and Their Application in Animal Liquid Feed

Chaima Ragoubi, Laura Quintieri, Donato Greco, Amel Mehrez, Imed Maatouk, Vito D'Ascanio, Ahmed Landoulsi and Giuseppina Avantaggiato

Table S1. Residual mycotoxin concentration (µg/mL) in supernatant samples obtained by incubating viable (VC) and heat inactivated cells (HIC) of *L. acidophilus* CIP: 76.13T and *L. delbrueckii* supp. *bulgaricus* CIP: 101027T with 1 µg/mL of each mycotoxin. Mycotoxin removal by LAB strains was determined in MRS and PBS after 24 h of incubation at 37 °C under aerobic conditions. AFB_1: Aflatoxin B_1, OTA: Ochratoxin A, ZEA: Zearalenone, DON: Deoxynivalenol.

Residual Mycotoxin Concentration in Supernatant Samples (µg/mL)								
	**ZEA**		**DON**		**AFB_1**		**OTA**	
**LAB Strain**	MRS	PBS	MRS	PBS	MRS	PBS	MRS	PBS
VC *L. acidophilus* CIP: 76.13T	0.71 ± 0.17	0.43 ± 0.09	0.91 ± 0.06	0.69 ± 0.13	0.67 ± 0.16	094 ± 0.14	0.95 ± 0.29	0.98 ± 0.68
VC *L. delbrueckii* ssp. *bulgaricus CIP: 101027T*	0.71 ± 0.21	0.44 ± 0.09	0.95 ± 0.04	0.70 ± 0.02	0.69 ± 0.18	0.84 ± 0.07	0.85 ± 0.13	0.96 ± 0.06[d]
HIC *L. acidophilus* CIP: 76.13T	0.88 ± 0.21	0.65 ± 1.13	0.97 ± 0.04	0.86 ± 0.06	0.69 ± 0.32	0.88 ± 0.12	0.98 ± 0.13	0.88 ± 0.40
HIC *L. delbrueckii* ssp. *bulgaricus CIP: 101027T*	0.89 ± 0.19	0.88 ± 0.09	0.66 ± 0.14	0.81 ± 0.24	0.72 ± 0.47	0.86 ± 0.12	1.01 ± 0.13	0.82 ± 0.54

Table S2. Residual ZEA concentration in liquid feed (LF) obtained incubating viable cells of L. acidophilus CIP: 76.13T and L. delbrueckii supp. bulgaricus CIP: 101027T with 1 µg/mL of the toxin. ZEA removal in LF by LAB strains was determined after 24 and 48 h of incubation in LF at 37 °C. Microbial count at 0, 24 and 48 h is also reported. Values represent means ± standard deviations of three independent experiments.

**Strain**	**ZEA Concentration (µg/mL)**		**Log cfu/mL**		
	24h	48h	0h	24h	48h
VC *L. acidophilus* CIP: 76.13T	0.86 ± 0.01	0.78 ± 0.02	5.6 ± 0.1	7.5 ± 0.1	5.6 ± 0.8
VC *L. delbrueckii ssp. bulgaricus* CIP: 101027T	0.89 ± 0.02	0.77 ± 0.02	5.8 ± 0.1	9.5 ±0.6	8.9 ± 1.1
